# The mystery of the fourth beat

**DOI:** 10.1007/s12471-015-0791-5

**Published:** 2016-01-13

**Authors:** F. van den Brink, I. Frenaij, R. van Tooren

**Affiliations:** St Antonius Hospital, Nieuwegein, The Netherlands

The ECG (see fig. 1 in the Question) shows a sinus rhythm of 97 beats per minute, a normal axis, a small QRS complex and only mild ST depression in all leads with slight elevation in aVR. Furthermore it shows a 4:1 ratio electrical alternans. During CPR, the patient suffered 3 left-sided and 5 right-sided rib fractures. As breathing caused negative intrathoracic pressure, the sternum collapsed shifting the heart to the left creating the 4:1 ratio electrical alternans. On intubation, a positive intrathoracic pressure was created causing the sternum to remain stable and the electrical alternans to disappear (Fig. 2). Although no definite cause for her out-of-hospital cardiac arrest has yet been found, a hereditary disease seems likely as her mother died suddenly of unknown cause at the same age. The patient made a full recovery.

**Fig. 2:**
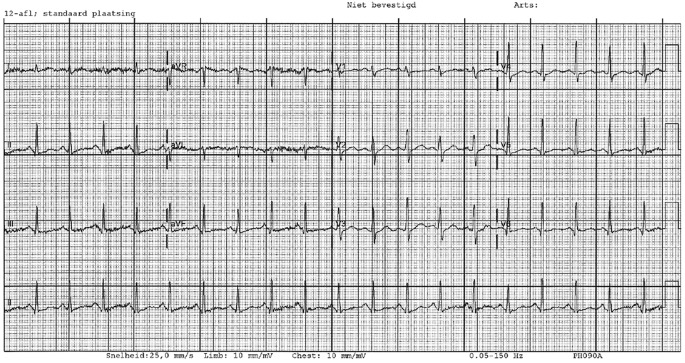
ECG after intubation

## Funding

None.

## Conflict of interest

None declared.

